# Measures to Improve Influenza Vaccination Coverage in Spanish Medical Students

**DOI:** 10.3390/vaccines8020238

**Published:** 2020-05-20

**Authors:** Ignacio Hernández-García, Carlos Aibar-Remón

**Affiliations:** 1 Department of Preventive Medicine, Lozano Blesa University Clinical Hospital of Zaragoza, Calle San Juan Bosco 15, 50009 Zaragoza, Spain; caibaremon@gmail.com; 2 Health Services Research Group of Aragon (GRISSA), Aragon Institute for Health Research (IISA), Calle San Juan Bosco 15, 50009 Zaragoza, Spain; 3 Department of Preventive Medicine and Public Health, University of Zaragoza, Calle de Pedro Cerbuna 12, 50009 Zaragoza, Spain

**Keywords:** Students, medical, influenza vaccines, vaccination coverage, strategies, Spain

## Abstract

*Objective*: To find out what measures medical students believe could help improve their influenza vaccination coverage. *Method*: On 5 November 2019, the Dean of the Zaragoza Medical School sent an e-mail to the students asking them to fill out a questionnaire through Google Forms, in which they were asked to describe, in an open field, the measures that they believed could contribute to improving their flu vaccination coverage. The content of the responses was analyzed in a classic way, extracting descriptors and selecting the most representative verbatim accounts. *Results*: The main measures proposed were to improve the training on influenza and its vaccine, to improve the accessibility of the vaccine in time and space, to provide incentives to get vaccinated, to create visible and positive attitudes towards the vaccine, and to increase the diffusion of information about the vaccination campaign. *Conclusion*: This qualitative study has found potential measures to be applied specifically to medical students to improve their vaccination coverage in our country.

## 1. Introduction

Healthcare workers (HCWs) are particularly exposed to vaccine-preventable diseases and play a major role in nosocomial transmission, which makes them an important target group for vaccination. Vaccination recommendations in HCWs differ according to country. Hepatitis B, pertussis, and measles vaccines are usually recommended for all HCWs in high-income countries [1]. Influenza vaccination is recommended for all HCWs in 24 European countries, the USA, and Japan [1,2].

Influenza vaccination of HCWs is one of the most effective measures to avoid nosocomial outbreaks. Other benefits obtained from vaccination of HCWs are self-protection, decreased absenteeism rates, and a lower rate of morbidity and mortality among their patients [3]. However, in Europe, the coverage among HCWs ranges from 9.7% in Slovenia to 54.9% in the United Kingdom [2]. In the USA, the coverage ranges from 47.6% among those working in settings where vaccination is not required to 94.8% among HCWs who were required by their employer to be vaccinated [4]. In Japan, influenza vaccination coverage among HCWs is as high as 85.0% [5].

Influenza vaccination among trainee students in healthcare settings is recommended to reduce the risk of them acquiring influenza and transmitting it to patients [6,7]. In particular, the vaccination coverage in medical students is low [8,9,10,11,12,13], with rates of between 16.5% [8] and 68.9% [12] in Polish and North American students, respectively. In Spain, the coverage rate for influenza vaccination among HCWs is 35.0% [14], and among medical students, it is 5.9% [15]. For this reason, it is necessary to consider specific improvement interventions for this group, as recently formulated for pregnant women and health workers [16].

This research was carried out in order to find out what measures could contribute to improving vaccination coverage in medical students.

## 2. Material and Methods

A qualitative study was performed at the University of Zaragoza. Students enrolled in the third to sixth year of medical school were included. To obtain the information, on 5 November, 2019, the Dean of the School sent an e-mail to the students asking them to fill out a questionnaire through Google Forms. This questionnaire (which was not previously validated) had only one question asking participants to describe, using an open field, the measures that they believed could contribute to improving their flu vaccination coverage. The questionnaire informed participants about the objective of the study, as well as the voluntary, confidential, and anonymous nature of the study.

The content of the responses was analyzed in a classical way [17], extracting descriptors and selecting the most representative verbatim accounts; in particular, the descriptors were coded by identifying the main information and assigning labels to group them.

## 3. Results

A total of 5.5% (46/836) of the students answered the questionnaire. The number of improvement measures proposed was 98. The following seven descriptors were extracted (Table 1).

### 3.1. Influenza Training

Eight people reflected on the need for more flu training: “Give more information on the overwhelming data on deaths and complications due to the flu” (student 31) and “Show the effects that not getting vaccinated can have on the patient” (student 30).

### 3.2. Flu Vaccination Training

Nineteen students referred to improving influenza vaccine training: “More information on the importance and effectiveness of vaccination” (student 23) and “More information on vaccine safety and effectiveness […]” (student 22). In particular, 10 students reported improved training on the reasons for vaccination, especially self-protection and protecting patients.

### 3.3. Access to Vaccination

Improving the accessibility of the vaccine, in terms of time and space, was the descriptor that brought together the largest number of proposals (23 students made 30 proposals): “Vaccinate at the school itself” (student 34) and “Facilitate vaccination on a morning and afternoon schedule […]” (student 9).

### 3.4. Incentives or the Obligatory Nature of Vaccination

Four students suggested that they should be required to get vaccinated or receive incentives to do so: “Obligatory to be able to do the internship” (student 20) and “Give 0.5 credits to the student who gets vaccinated” (student 46).

### 3.5. Increased Visibility of Positive Attitudes towards Vaccination

Seven people proposed increasing the visibility of positive attitudes towards vaccination: “Raise awareness among practice tutors so that they also get vaccinated and set an example […]” (student 30), “Badges like the ones being given this year to everyone who gets vaccinated (it seems silly, but the fact that many people get vaccinated creates group awareness and the badge makes it more visible)” (student 31), and “More recommended by teachers” (student 36).

### 3.6. New Technologies to Inform Students about the Convenience of Vaccination

Four students suggested sending emails and using social networks to spread the importance of vaccination: “Social networking and communication to delegates to spread the idea about vaccination (people are more likely to listen to those they trust than strangers)” (student 38) and “Sending an informational email with everything you should know about vaccination” (student 17).

### 3.7. Information on the Vaccination Campaign

Fifteen people said they would spread more information about the vaccination campaign that includes students on training in health centers: “More posters by the university informing about the vaccination campaign” (student 20) and “Announcing the campaign in class and on the advertising screen” (student 21).

## 4. Discussion

This paper is the first to analyze the opinions of medical students on how to improve influenza vaccination coverage in this group. This is relevant because, in the design of the strategies proposed so far to increase influenza vaccination rates in Spain, no specific measures have been considered for this target group [16], perhaps because of the assumption that the strategies for health students are the same as those for health professionals [18].

However, according to our findings, this assumption is not plausible. For example, students suggested, as an important measure for improvement, that vaccination should be done at the medical school itself (in the building where they receive their theoretical classes) rather than at the healthcare setting where they practice (which is where health workers are advised to be vaccinated [16]).

On the other hand, the fact that students suggested receiving more training on influenza and its vaccine is consistent with what has been found in other studies carried out in our environment, in which a level of knowledge that can be improved upon regarding this vaccine has been observed in medical students [19]. This finding is especially important given that a better level of knowledge has been associated with a higher frequency of vaccination [12] and that the years of careers are the ideal time to ensure students’ understanding through exam evaluation [19].

Other proposed measures are consistent with strategies that have been observed to improve vaccination uptake rate among HCWs (such as the role models of senior HCWs receiving vaccination, mandatory vaccination polices, declination statements, increased access, and increased awareness) [4,20,21,22]. The impact of implementing the above proposals (with the exception of obligatory vaccination, since in Spain this is not legally possible under existing laws), together with those on improving the dissemination of the publicity of the vaccination campaign, could be the subject of a quantitative large-scale study in the future.

Among the limitations of our study is the nonresponse bias, which is common in similar work (with response rates of 2.1% [23] and 9.1% [9]) and could be related to the lack of confidence in participating in online surveys [9], despite participants being informed of the confidentiality and anonymous nature of the research and the study not collecting variables that would allow for the identification of the student. Another limitation is that of cross-sectional studies, as well as the fact that our study was conducted in a single medical school. This makes it necessary to carry out similar studies to establish whether the proposed measures are the same in the rest of Spain, which is foreseeable given the similarity of the training programs and the means available (in Spain, the flu vaccine is free for health students).

With this study, we have found potential measures to improve flu vaccination coverage in medical students. This is relevant information because getting them into this habit can also mean that they continue to be vaccinated when they work as health workers, since a factor associated with vaccination is having been previously vaccinated [24].

## 5. Conclusions

This qualitative study has found potential measures to be applied specifically to medical students to improve their vaccination coverage in Spain.

## Figures and Tables

**Table 1:** Description of measures to improve vaccination coverage in medical students.

Responses	Descriptors	Theme
“Give more information on the overwhelming data on deaths and complications due to the flu” (student 31)	Influenza training	Measures to improve influenza vaccination coverage in medical students
“Show the effects that not getting vaccinated can have on the patient” (student 30)		
“Give more information about the importance of the flu” (student 3)		
“More information on the importance and effectiveness of vaccination” (student 23)	Flu vaccination Training	
“More information on vaccine safety and effectiveness […]” (student 22)		
“Show the vaccine as a method of protection for both self and patient” (student 5)		
“More information on the reasons for vaccination: protecting patients and the environment (student 4)		
“Vaccinate at the school itself” (student 34)	Access to vaccination	
“Facilitate vaccination on a morning and afternoon schedule […]” (student 9)		
“Giving time off to go for vaccinations” (student 28)		
“Vaccination in the university halls” (student 39)		
“Obligatory to be able to do the internship” (student 20)	Incentives/obligatory nature of vaccination	
“Proposing incentives for students” (student 23)		
“Badges like the ones being given this year to everyone who gets vaccinated (it seems silly, but the fact that many people get vaccinated creates group awareness and the badge makes it more visible)” (student 31)	Increase visibility of positive attitudes towards vaccination	
“Social networking and communication to delegates to spread the idea about vaccination (people are more likely to listen to those they trust than strangers)” (student 38)	New technologies to inform students about the convenience of vaccination	
“Sending an informational email with everything you should know about vaccination” (student 17)		
“More posters by the university informing about the vaccination campaign” (student 20)	Information on the vaccination campaign	
“Announcing the campaign in class and on the advertising screen” (student 21)		
“Send posters through WhatsApp to delegates to share with colleagues (better than large paragraphs of text that few people read to the end)” (student 12)		
“In addition, you could say in the classes so that it can be heard as well, since there are people who do not stop to read posters” (student 14)		
